# Evaluation of the TRMM product for monitoring drought over Paraíba State, northeastern Brazil: a trend analysis

**DOI:** 10.1038/s41598-020-80026-5

**Published:** 2021-01-13

**Authors:** Reginaldo Moura Brasil Neto, Celso Augusto Guimarães Santos, Jorge Flávio Casé Braga da Costa Silva, Richarde Marques da Silva, Carlos Antonio Costa dos Santos, Manoranjan Mishra

**Affiliations:** 1 Department of Civil Engineering and Environmental Engineering, Federal University of Paraíba, João Pessoa, 58051-900 Brazil; 2 Department of Geosciences, Federal University of Paraíba, João Pessoa, 58051-900 Brazil; 3 Academic Unit of Atmospheric Sciences, Federal University of Campina Grande, Campina Grande, 58109-970 Brazil; 4 Department of Natural Resource Management & Geoinformatics, Khallikote University, Berhampur, Ganjam, Odisha India

**Keywords:** Hydrology, Natural hazards

## Abstract

Droughts are complex natural phenomena that influence society's development in different aspects; therefore, monitoring their behavior and future trends is a useful task to assist the management of natural resources. In addition, the use of satellite-estimated rainfall data emerges as a promising tool to monitor these phenomena in large spatial domains. The Tropical Rainfall Measuring Mission (TRMM) products have been validated in several studies and stand out among the available products. Therefore, this work seeks to evaluate TRMM-estimated rainfall data's performance for monitoring the behavior and spatiotemporal trends of meteorological droughts over Paraíba State, based on the standardized precipitation index (SPI) from 1998 to 2017. Then, 78 rain gauge-measured and 187 TRMM-estimated rainfall time series were used, and trends of drought behavior, duration, and severity at eight time scales were evaluated using the Mann–Kendall and Sen tests. The results show that the TRMM-estimated rainfall data accurately captured the pattern of recent extreme rainfall events that occurred over Paraíba State. Drought events tend to be drier, longer-lasting, and more severe in most of the state. The greatest inconsistencies between the results obtained from rain gauge-measured and TRMM-estimated rainfall data are concentrated in the area closest to the coast. Furthermore, long-term drought trends are more pronounced than short-term drought, and the TRMM-estimated rainfall data correctly identified this pattern. Thus, TRMM-estimated rainfall data are a valuable source of data for identifying drought behavior and trends over much of the region.

## Introduction

Droughts, climate variability, changes in land use and land cover, and the lack of formal policies on water resources are the main factors affecting water availability in northeastern Brazil (NEB)^1^. Meteorological drought is a natural phenomenon caused by the absence of rainfall over a certain period and can damage the development of society's different activities^2^. Currently, problems resulting from droughts and water scarcity are increasingly recurrent in the daily lives of a large part of the population living in the Brazilian semiarid region (BSAR) and affect the social and economic development of that region^3–5^. Among the causes that contribute to this situation, the rainfall irregularity and the water storage problem in large, medium, and small cities in the BSAR stand out. Furthermore, the high rates of evaporation and the increased demand for water by the population are also other factors that contribute to this scenario.

Monitoring meteorological droughts and their effects is not an easy task. It is necessary to have a monitoring network that accurately captures the spatiotemporal rainfall variability, which is a challenge, especially in the arid and semiarid regions^6^. In addition to the rainfall variability, it should be noted that although longer rainfall time series are usually obtained from rain gauges^7^, in tropical and developing regions, the absence of financial and technical resources results in networks of poorly distributed rain gauges or in failures in the time series hindering the monitoring of these natural disasters. Thus, orbital remote sensing-estimated rainfall data emerge as alternative data sources for monitoring rainfall globally with high spatiotemporal resolution^8^. With the evolution of technology, these estimates have been used to monitor natural disasters and assist in managing water resources in large areas and areas with a lack of meteorological data^9^.

In this context, the Tropical Rainfall Measuring Mission (TRMM) rainfall estimated data had been widely evaluated and used satisfactorily to monitor drought patterns in different regions^10–18^. The use of TRMM in the context of drought assessment based on multiple time scales is of significant interest due to the possibility for monitoring this phenomenon and its different impacts. Some studies^14,16^ indicated that TRMM-estimated data perform better in identifying short-term drought, whereas other studies showed that the performance is better for long-term droughts^13,15^. In some cases, there is not a major difference in the performance of TRMM-estimated data to monitor short-, medium- and long-term drought patterns^11,17^. These different results are due to the physical characteristics of the regions, the length of the time series used and the number of rain gauges used to calibrate the TRMM estimates.

Combined with the ability to characterize these natural disasters on a large spatial scale and a refined temporal scale, the analysis of drought trends is another theme that has attracted the attention of several studies. This is closely related to the fact that the future impacts of climate change for semiarid regions can cause the intensification and prolongation of droughts and generate serious problems such as water scarcity and water supply collapse. In addition, droughts can create socio-environmental impacts such as desertification, reduced agricultural potential, and rural exodus to urban areas^19^. For this reason, assessing drought trends and their characteristics based on different statistical methods, such as Mann–Kendall and Sen non-parametric tests, is a practice that has contributed actively to improve the efficient management of available resources in different regions^20–28^.

Among the indices commonly used to monitor droughts and their effects, the standardized precipitation index (SPI)^29^ is considered one of the most widespread in the world. The SPI is a standardized index that allows the evaluation of droughts at various time scales and severity categories, enabling comparisons among different regions, requiring only rainfall data to be computed, facilitating the SPI application compared to other more complex indices. Each drought index has different characteristics, which are suitable for specific environments^30^, stimulating several comparisons of different indices in the literature in different climatic regions of the planet^31–33^.

It is important to expose an extremely high limitation of long-term and high-quality hydrometeorological time series in arid and semiarid regions worldwide, which is even more relevant considering such regions in developing countries, which is the case of Paraíba State. For this reason, evaluate the drought pattern using indices that involve more variables, as is the case of SPEI^34,35^, is difficult. On the other hand, the use of SPI is a crucial tool to assess the geospatial distribution of droughts over a region, and this index has satisfactorily been used to monitor droughts at multiple time scales in NEB and Paraíba State^5,18,36–39^.

Paraíba State is formed by four physiographic mesoregions distributed in different biomes. Mata Paraibana is located in the Atlantic Forest biome, the Borborema and Sertão Paraibano mesoregions in the Caatinga biome, and Agreste Paraibano is a transition zone^40^. These mesoregions have different climatic characteristics, and understanding droughts' behavior in areas with contrasting characteristics is of great relevance for managing the water resources in Paraíba State^41^. Furthermore, such mesoregions have climatic and relief patterns that influence vegetation, soil types, rainfall variability, and water availability^42^.

In Paraíba State, Brasil Neto et al.^18^ showed that TRMM is more accurate in identifying the pattern of medium-term droughts, but the results vary according to the performance index used. It is worth highlighting that except for the study proposed by Brasil Neto et al.^18^, we are not aware of any other research that evaluated the performance of TRMM product in the context of multitemporal monitoring of meteorological droughts or in comparing drought trends based on satellite-estimated and rain gauge-measured rainfall data. However, the environmental impacts of frequent drought episodes in Brazil have resulted in drought-related studies^43^, and interest in regional assessments of droughts is emerging as part of novel management initiatives to build drought resilience^44^. Finally, this study aims to assess the TRMM-estimated rainfall data’s performance in monitoring meteorological droughts’ behavior and trends at multiple time scales based on the SPI over Paraíba State for the past 20 years (1998–2017).

## Materials and methods

### Study area

**Figure 1:**
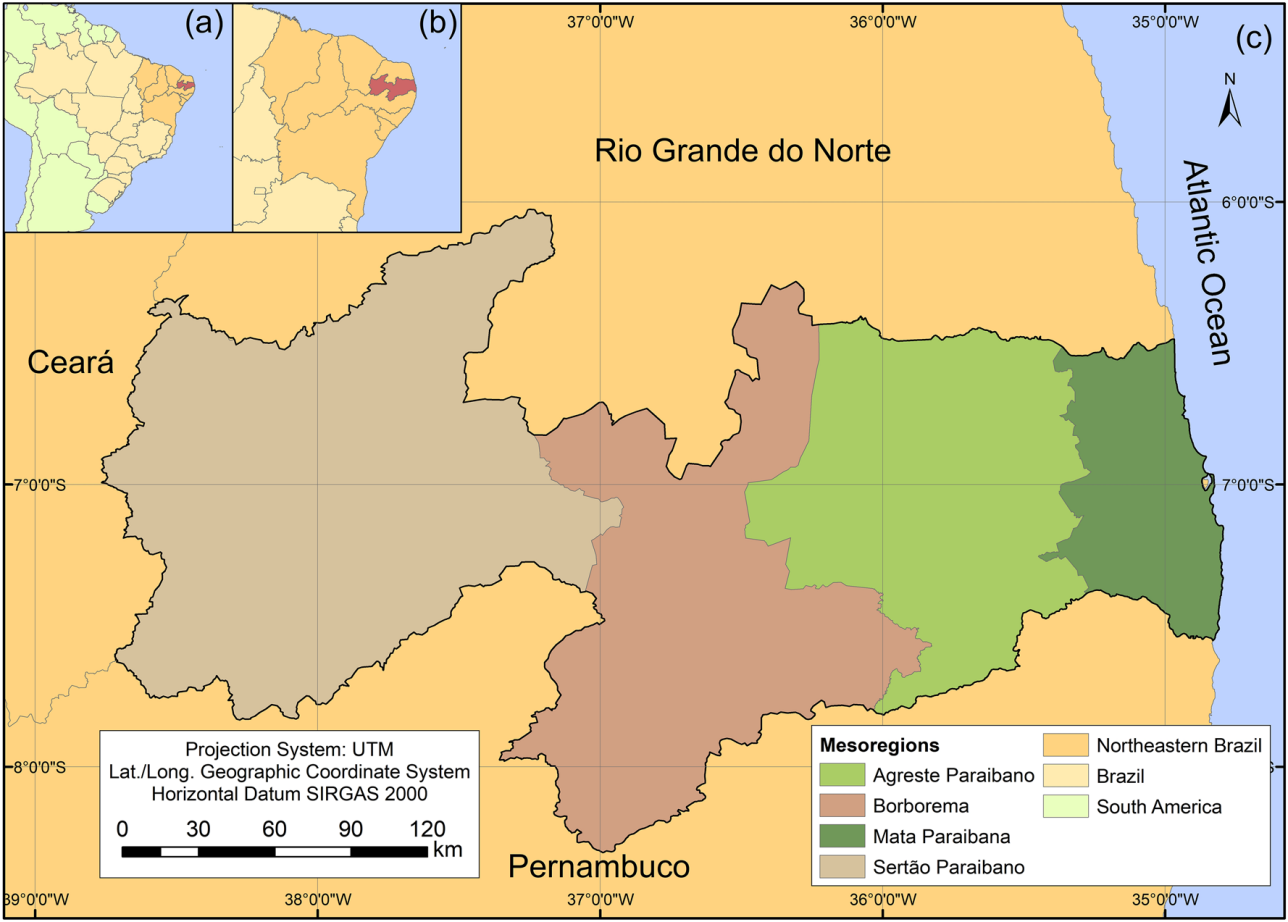
Location of mesoregions in Paraíba State, northeastern Brazil.

The study area is Paraíba State, limited by latitudes 5.875° S and 8.375° S, and longitudes 38.875° W and 34.625° W. Paraíba State has an area of 56,469.78 km^2^ and is bordered by Ceará State (to the west), Rio Grande do Norte State (to the north), the Atlantic Ocean (to the east), and Pernambuco State (to the south). Paraíba State has a population of approximately four million inhabitants distributed in 223 municipalities; the state is divided into the aforementioned four mesoregions^45^ (Fig. 1). According to the physical characteristics, Paraíba State is formed by two biomes: Atlantic Forest and Caatinga. The Atlantic Forest biome covers not only the ridge but the coastal plains. This biome is well defined in Paraíba State and covers an area of 6578 km^2^. This area has climates with high air relative humidity and abundant and well-distributed rainfall throughout the year. These features support predominantly forested vegetation, known as Atlantic Forest, which shelters a rich and threatened endemic biological diversity^46^. The Caatinga biome includes two markedly different climatic sectors. The rainfall seasonality and the controlling factors, such as drought frequency and length, are distinct between these two areas^47^. Details on the characteristics of the region can be obtained from Santos et al.^42^ and Santos et al.^39^.

### Rainfall data

#### Rain gauge-measured rainfall data

A total of 251 daily rainfall time series were obtained from rain gauges distributed throughout the study area, from 1998 to 2017, available from the Water Management Executive Agency (AESA). Daily data were accumulated at a monthly level to calculate the SPI and to develop the drought analysis. However, time series with missing data were excluded from the following analyses, resulting in 78 complete time series to be used in this study. The accuracy of the TRMM-estimated rainfall data in capturing the behavior of droughts was assessed, using only complete time series to avoid filling the gaps, which could distort the results and expose the situation of the region regarding the availability of data. Although we have known that it is better to use the longest time series as possible since the results must be more reliable, extend the period of our study to 30 years will imply in decrease the number of rain gauge time series used as reference, which is not a good solution, because we are using only complete time series. More details regarding the qualitative analysis of the rainfall data available in the region can be found in Brasil Neto et al.^18^.

#### Satellite-estimated rainfall data

To carry out drought monitoring, complete and equally distributed TRMM-estimated rainfall data over Paraíba State were used^48^. TRMM was a research satellite designed to monitor rainfall within the tropics^49^. Among the available products, the TRMM Multi-satellite Precipitation Analysis (TMPA) (https://giovanni.gsfc.nasa.gov/giovanni/) is the one that combines remote sensing estimated precipitation data with the rain gauge and radar precipitation observations when these observations are available^50^. TMPA products are capable of covering extensive space domains, i.e., between latitudes 50° N and 50° S and longitudes 180° W and 180° E. The data have a refined spatial resolution of 0.25° × 0.25°, allowing rainfall monitoring in various areas of the globe.

**Figure 2:**
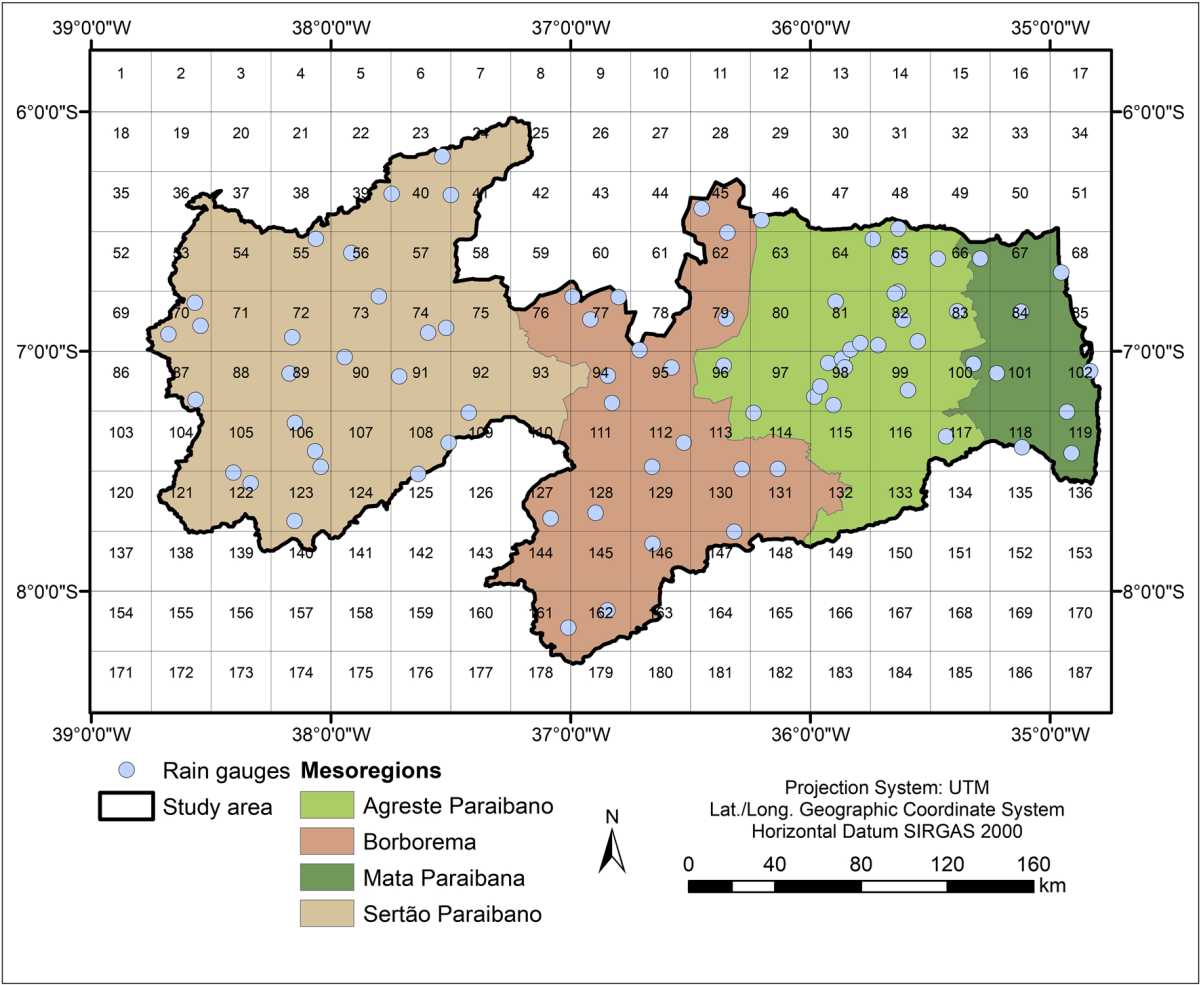
Spatial distribution of the TRMM grid and the rain gauges used over Paraíba State.

In Paraíba State, studies used the TMPA estimates, and the results indicate that these estimates are quite viable data sources to capture the rainfall patterns and meteorological droughts^18,39,42,51^. Daily data from TMPA 3B42v7 were used in this work, and the study area was divided into 187 grids (11 × 17). Figure 2 shows the spatial distribution of the TRMM grid and the rain gauges used in this study. Daily rainfall time series were accumulated monthly from January 1998 to December 2017, obtaining slightly less than 45,000 data of accumulated monthly totals (187 TRMM series × 20 years × 12 months).

### Standardized precipitation index and run theory

The SPI calculation was based on the adequacy of the rainfall data to a gamma distribution of α and β parameters, and eight SPI values were used to monitor droughts at multiple time scales: SPI-1, SPI-3 and SPI-6 for short-term droughts; SPI-9 and SPI-12 for medium-term droughts; and SPI-18, SPI-24 and SPI-48 for long-term droughts. The period used to compute the SPI values was from January 1998 to December 2017. All SPI values for each time scale and available series, i.e., 78 rain gauge-measured time series provided by AESA and 187 TRMM-estimated rainfall time series, were calculated. In addition, four severity categories were used to classify dry and wet events. The dry events are those whose SPI values are less than or equal to zero, and the wet events are those with SPI greater than zero.

**Figure 3:**
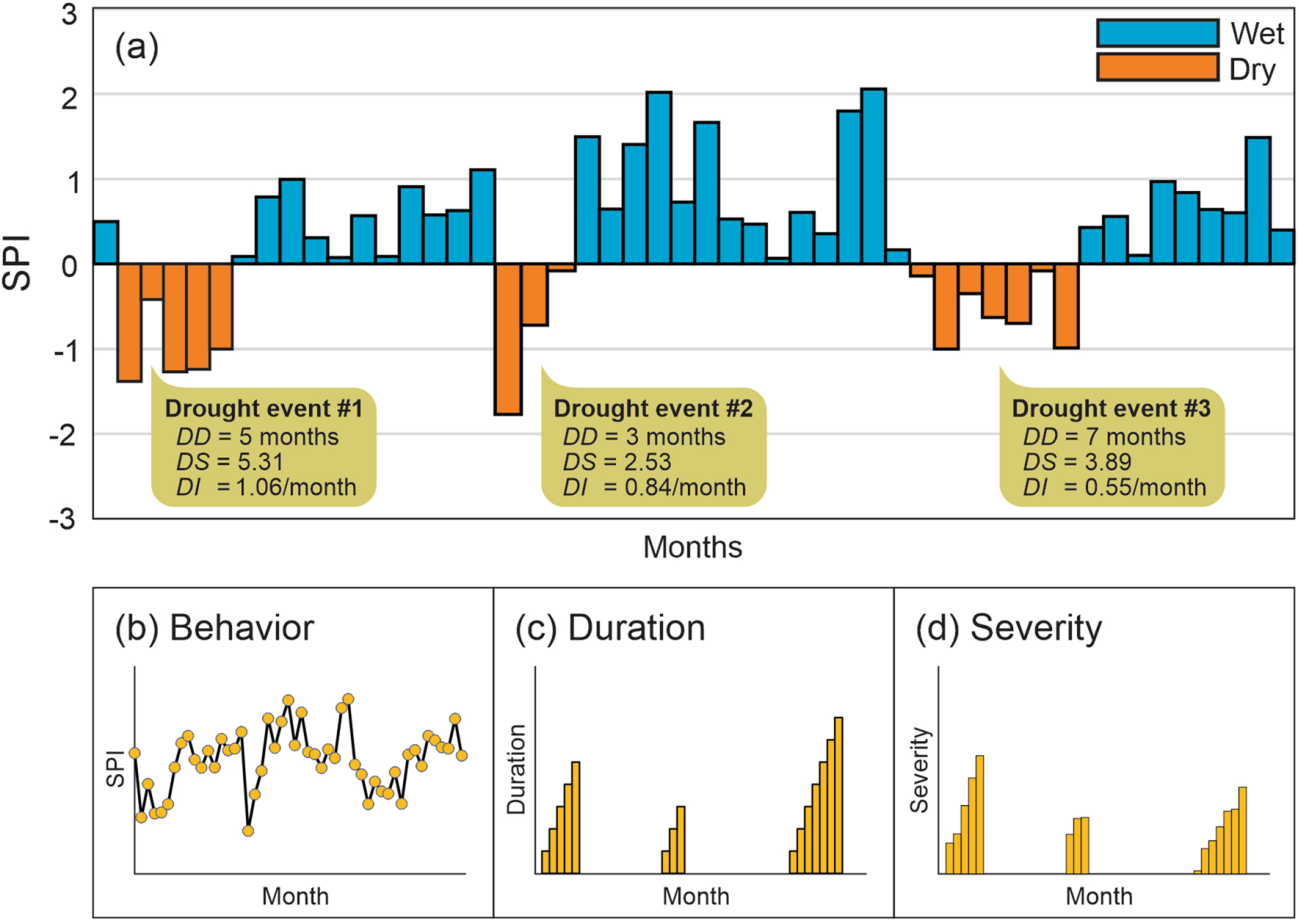
Definition of (**a**) drought events, and illustration of the time series of drought (**b**) behavior, (**c**) duration and (**d**) severity.

The categories related to the severity of events vary according to the SPI value, such as mild events (0.0 <|SPI|≤ 1.0), moderate events (1.0 <|SPI|≤ 1.5), severe events (1.5 <|SPI|≤ 2.0) and extreme events (2.0 <|SPI|). More details regarding the calculation of the SPI can be found in Santos et al.^52^. Furthermore, it was admitted that a drought event is characterized by the period in which exists continuity of dry events (SPI ≤ 0) according to the premise of Run Theory^53^. Based on this definition, three distinct drought time series were defined and used to assess the drought behavior, duration and severity, and trends at different time scales. Figure 3 depicts an example of three distinct drought events and the time series evaluated in this research.

The drought duration (DD) is the number of months between the start and end period of the drought events, the drought severity (DS) is the cumulative SPI values for the event, and drough intensity (DI) is the ratio between drought severity and duration. From Fig. 3, three distinct drought events stand out with durations of five, three and seven months, and it can be seen that the three events differ in terms of severity. Three types of time series were evaluated: (a) the behavior time series, composed of SPI values, (b) duration time series (DDS), consisting of the duration values of each drought event, and (c) the severity time series (DSS), consisting of the severity values of each event. Based on Fig. 3, the drought behavior time series is composed of 50 values that usually range from − 3 to 3, the DDS time series is composed of three duration values (i.e., 5, 3 and 7 months), and the DSS time series is composed by three severity values (i.e., 5.31, 2.53 and 3.89). Therefore, all drought metrics (DD, DS, DI) and drought time series (behavior, DDS, DSS) were computed based on these time series (8 scales × 265 times series). Moreover, our results were interpolated to improve the visualization of the maps, but we assessed all available values (time series) for each database (point and grid).

### Trend analysis

Analyses were developed to assess trends in short-, medium- and long-term drought time series and determine the respective magnitudes. Thus, three time series that reflect important drought characteristics were evaluated, and two non-parametric tests were chosen to carry out the trend analysis. The trends in the behavior (SPI), duration (DDS) and severity (DSS) time series (Fig. 3) were evaluated based on the Mann–Kendall^54,55^ and Sen^56^ tests. The Mann–Kendall test was used to identify a statistically significant trend, and the Sen test was used to estimate the linear magnitude of the identified trends.

The variability of the behavior time series calculated from the Mann–Kendall and Sen tests' application was evaluated monthly. In contrast, for the duration and severity time series, the variation was assessed based on events. For the behavior series, negative trends characterize the worst scenario and indicate that SPI values tend to be more negatively accentuated over the months. Still, for drought duration and severity time series, positive trends point to the worst scenario and indicate that drought events tend to be more lasting and severe for each event.

In this study, three levels of α significance were used (i.e., 0.01, 0.05 and 0.10), and analyses at single-gauge for each mesoregion were performed considering rain gauge-measured and TRMM-estimated rainfall data. In the mesoregional levels, Thiessen-method polygons of the rain gauge-measured and TRMM-estimated data were computed, and the percentage of area that presented significant trend was calculated. We highlight that the existence of autocorrelation between time series should be considered, which is why some researchers are concerned with removing the effects of autocorrelation between time series, as is done by the modified Mann–Kendall test (MMK). On the other hand, some studies do not consider such an autocorrelation or indicate that the results obtained using the MK or MMK tests are not so different^57,58^. Due to the similarity of Paraíba State with those regions, we did not use the MMK and assumed that this might not affect the results. More than 6,000 trend analyses were developed (265 rainfall time series × 8 SPI indices × 3 time series), and more details regarding the Mann–Kendall and Sen tests can be found in Santos et al.^39^ and Santos et al.^59^.

## Results and discussion

### Evaluation of rainfall and drought over Paraíba State

**Figure 4:**
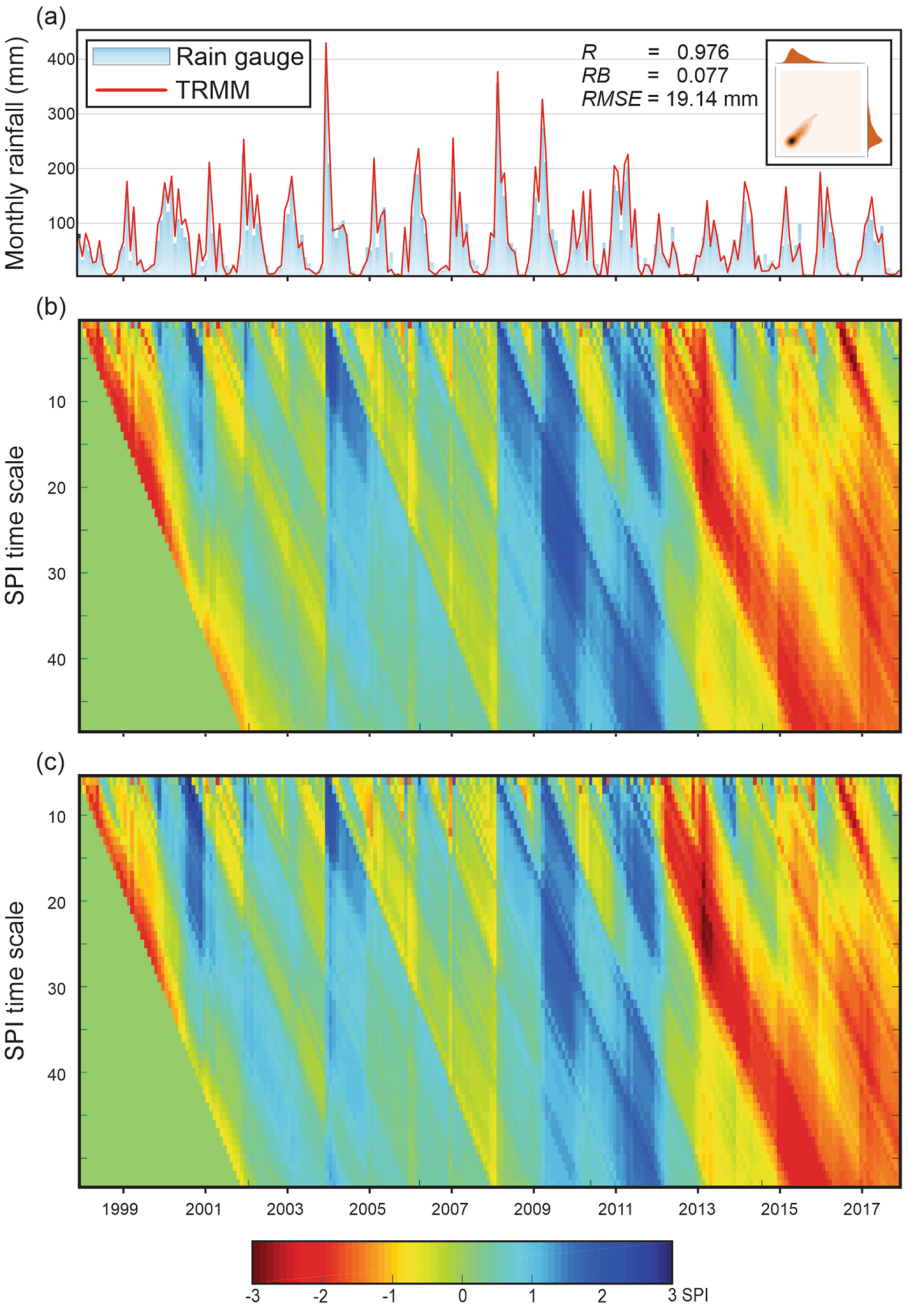
(**a**) Hyetograph and two-dimensional kernel density estimate plot (**Inset**) of monthly mean rainfall over Paraíba State using rain gauge-measured and TRMM-estimated rainfall data, and behavior of SPI-1 to SPI-48 based on (**b**) rain gauge-measured and (**c**) TRMM-estimated rainfall data (1998–2017).

Initially, the mean rainfall values over Paraíba State were obtained by taking Thiessen-weighted averages of the rain gauge-measured and TRMM-estimated rainfall data from 1998 to 2017 (Fig. 4a). The results show that the pattern of the rainfall time series is remarkably similar over the analyzed period. The metrics indicate the TRMM-estimated rainfall data's satisfactory performance to capture the regional rainfall regime (*R* = 0.98), which means an almost perfect linear association between these time series. From the relative bias (RB), which is the ratio between the difference between TRMM-estimated and rain gauge-measured rainfall data by the rain gauge-measured rainfall data, there is evidence that the TRMM-estimated rainfall data slightly overestimated the rain gauge-measured values (RB = 8%), which can be considered good, as Paraíba State is an extensive longitudinal region with different climates. The root mean squared error (RMSE) values were also low, which shows the accuracy of the TRMM-estimated rainfall data to monitor the regional rainfall behavior, as found by Pereira et al.^60^, Melo et al.^61^ and Soares et al.^51^.

These results can be related to many factors, such as the number of rain gauges, the precipitation pattern and the presence of circulation mechanisms over the region, as stated by McCollum et al.^62^. The number of rain gauges and their spatial distribution over Paraíba State must have affected the precision of the results, since there are regions that present very few rain gauges, making the comparison between the rain gauge-measured and TRMM-estimated rainfall data a challenging task. Moreover, the contrast between the precipitation pattern in inland and coastal zones and the existence of circulation mechanisms related to rainfall anomalies over NEB can contribute to the discrepancies between the datasets because of the humidity gradient^63,64^. A detailed analysis of the TRMM-estimated rainfall data's accuracy for monitoring droughts over Paraíba State can be found in^18^.

TRMM-estimated rainfall data precisely identified the rainy (e.g., 2004, 2008 and 2009) and dry (e.g., 1998 and 2012) years when compared to the mean annual rainfall levels within the region (1998–2017). However, despite the similarity between the time series, some overestimation and underestimation of the TRMM-estimated rainfall data are highlighted. In most cases, overestimations occurred in the rainy months, when the monthly totals of rainfall measured in the rain gauges were greater than 150 mm. On the other hand, TRMM-estimated rainfall data were underestimated when monthly rainfall was less than 50 mm (Fig. 4a). This result demonstrates the sensor’s inaccuracy when estimating the magnitude of precipitation. This may be related to the spatial scale of the TRMM-estimated rainfall data or due to the variability of rainfall within the region, because we are comparing single-gauge level results with the results at a grid level.

For instance, Pombo and de Oliveira^65^ compared the estimates of annual maximum daily precipitation from TRMM 3B42 to in-situ observation data in Angola, and the results show a slight underestimation. Prakash et al*.*^66^ reported limitations in estimating heavy rainfall over northern India and southeast peninsular India. Fang et al.^67^ showed that the satellite products capture the spatial patterns in extreme precipitation with relative accuracy, but less accurately estimate rainfall rates and volumes. The difference in accuracy at monthly scale may be due to the deviations caused by complex topography, rainfall rate, uncertainty of the rain gauges and their low density, which cannot accurately reflect the rainfall patterns within the areas^68,69^.

Then, the SPI time series at 48 time scales (from SPI-1 to SPI-48) were calculated based on rain gauge-measured (Fig. 4b) and TRMM-estimated (Fig. 4c) rainfall data from 1998 to 2017. The results show similarity in the obtained drought pattern, regardless of the temporal scale. It is possible to observe the sensor’s accuracy in capturing the drought regime over Paraíba State was satisfactory. In addition, from the SPI application, it is possible to identify more precisely the dry and wet periods and how wet or dry the events are over the period. It is worth noting that there is a pattern of similarity between the results, which shows well-defined vertical lines demarcating the beginning of the wet (e.g., 2004) and dry (e.g., 2012) periods. From that point, there is a change in the SPI behavior, and these values tend to be positive or negative in a diagonally downward direction, as found by Tan et al.^16^ and Yang et al.^70^.

However, there are some divergences between the SPI values calculated based on these time series. From 1998 to 2001, SPI values calculated based on satellite-estimated rainfall data (SPI_TRMM_) overestimated those obtained from rain gauge-measured rainfall data (SPI_gauge_). In other words, this means that the TRMM-estimated rainfall data estimated wetter events than rain gauge-measured rainfall data. The results show that after 2008, SPI_TRMM_ values were underestimated in relation to SPI_gauge_ values, regardless of the time scale. After 2012, which marks the beginning of one of the most severe drought events of recent times, there is an increase in the number of SPI_TRMM_ less than SPIgauge values (underestimation), especially when evaluating large time scales. The most considerable inconsistency between the two time series occurred in the case of short-term droughts. This may be linked to the fact that, for these time scales, few months were considered for computing the accumulated precipitation, unlike the SPI-48. Despite the occasional divergences, the results are valuable in the process of characterizing droughts, and the SPI_TRMM_ values captured drought events at multiple time scales over the study area during the 20 years studied.

**Figure 5:**
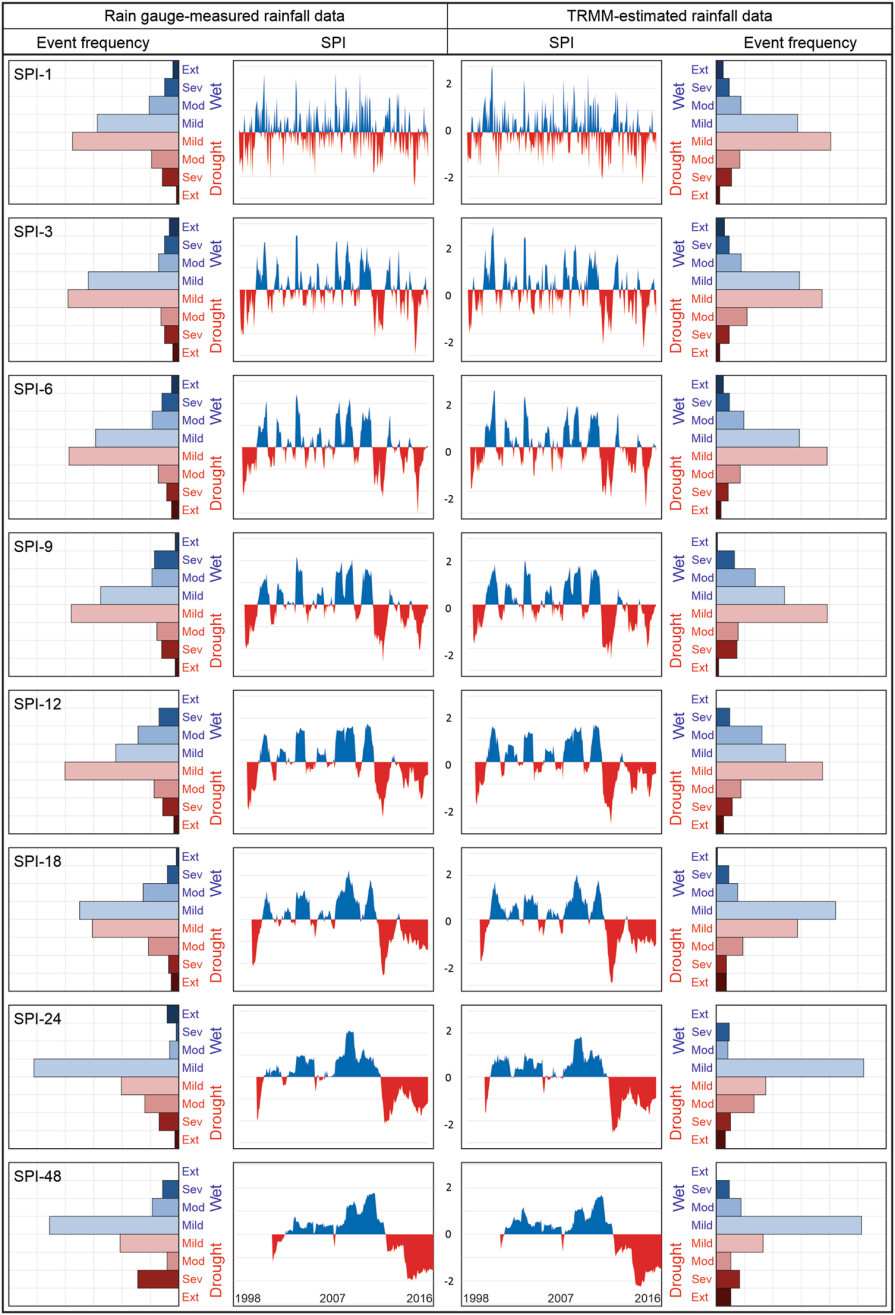
SPI_gauge_ and SPI_TRMM_ time series and frequency of the types of wet and dry events at multiple time scales based on the mean rainfall time series over Paraíba State.

Figure 5 shows the time evolution of the eight SPI_gauge_ and SPI_TRMM_ time series and the percentage of occurrence of each type of wet and dry event over the period. From Fig. 5, it can be seen that for short-term droughts, the SPI values often vary between positive and negative over time, i.e., there is a discontinuity between dry and wet events, as found by Santos et al.^52^. Although the SPI values at smaller time scales are sensitive to the occurrence of extreme rainfall events, the SPI_TRMM_ and SPI_gauge_ values were still remarkably similar to each other. Regarding the frequency of events, the results indicate greater similarity between the percentages of mild dry events. For SPI-1, about 55% of the events were dry, but for SPI-3 and SPI-6, this percentage was slightly lower. The results of SPI_TRMM_ sometimes overestimated SPI_gauge_ results (e.g., between 1998 and 2001), and sometimes underestimated them (e.g., from 2008). Among the most notable errors on the part of TRMM-estimated rainfall data, the overestimation of moderate dry events by TRMM-estimated rainfall data stands out when evaluating the SPI-3.

Unlike the behavior of short-term droughts, it is noted that there is a temporal continuity of dry and wet events for medium-term droughts, which facilitates the identification of wet and dry periods, as defined by Wable et al.^71^. From the results of SPI-9 and SPI-12, it can be noted that 2004, 2008 and 2009 were the wettest years, while 1998, 1999 and 2012 were the driest years in the entire period. Despite the similarity between the SPI_gauge_ and SPI_TRMM_ series, it is noted that there are still divergences on the part of SPI_TRMM_, which overestimated the values in 2002. From the percentage of occurrence of the events, the similarity in the pattern of the results was more evident than in the case of short-term droughts.

The percentage of dry events increased, mainly in the case of SPI_gauge_ results. For SPI-12, the total percentage of dry events was 56%, based on SPI_gauge_ and SPI_TRMM_, reflecting an increase of 3% compared to SPI-3 and SPI-6. The mild dry events were the most frequent in the analyzed period, a result analogous to that obtained for short-term droughts. On the other hand, almost none extreme wet events were registered when evaluating medium-term droughts, but moderate wet events were more recurrent. For SPI-9, the frequencies of severe and mild wet events were underestimated by SPI_TRMM_, while moderate wet events were overestimated. In the case of SPI-12, the percentage of severe wet events based on SPI_TRMM_ was underestimated, as well as for SPI-9 results.

Negative or positive SPI values tend to be even more continuous for long-term droughts than for short- and medium-term droughts. On the other hand, although long-term droughts' behavior has been identified, the results for SPI_TRMM_ have great variability. Until 2003, there was an overestimation of SPI_gauge_ for SPI-18 and SPI-24, but after 2012, there was an underestimation of the SPI-18, SPI-24, and SPI-48 values. In addition, compared to the results of short- and medium-term droughts, there is a change in the frequency of the event types. For SPI-18, SPI-24, and SPI-48, mild wet events were the most frequent events over the 20 years, and the frequency of dry events (SPI ≤ 0) has dropped considerably. For SPI-48, the percentage of dry events was 39% and 35% based on SPI_gauge_ and SPI_TRMM_, respectively, lower than that obtained for short- and medium-term droughts. The frequency of extreme and severe dry events, on the other hand, increased, and this result corroborates the pattern obtained in Santos et al.^39^, which also indicates that when evaluating larger SPI scales, the frequency of dry events decreases, but those events become more severe.

### Analysis of drought behavior trends

**Figure 6:**
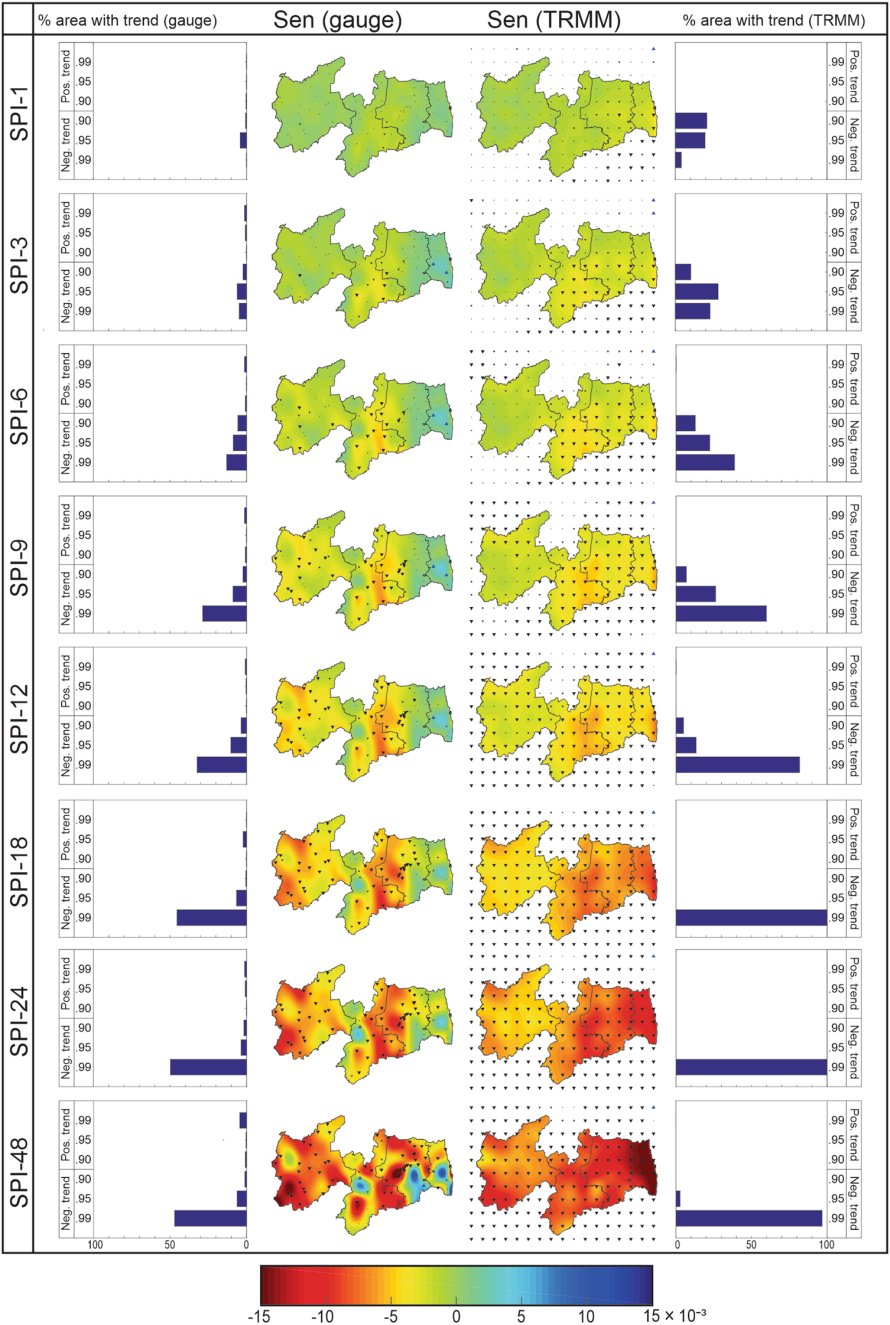
Percentage of the area with the significant trend and spatial distribution of the Sen’s slope of the drought behavior time series over Paraíba State (1998–2017).

Figure 6 shows the spatial distribution of the Sen’s slope of the drought behavior time series and the percentage of area with a significant trend based on different confidence levels over Paraíba State at various time scales. From this figure, it is noted that there is considerable variability among the results when analyzing the rainfall data and SPI time scales. The comparison between the SPI_gauge_ and SPI_TRMM_ results shows that Sen’s slope values increase as the time scale increases. The same happens when analyzing the percentage of areas with a significant trend. In other words, in the case of long-term droughts, the behavior time series has a steeper slope, with significant trends with a high degree regardless of the dataset used. This result is a pattern concerning the trend analysis of the drought behavior time series, as found in Asia^23^ and Oceania^72^.

When comparing the results obtained from the rain gauge-measured and TRMM-estimated rainfall data, the spatial variability related to both Sen’s slope and significance level is highlighted. There are areas where the results of the Mann–Kendall and Sen tests are consistent between both datasets, e.g., in Sertão and Borborema mesoregions when evaluating SPI-1, where the series of rain gauge-measured and TRMM-estimated rainfall data presented mildly negative Sen’s slopes and showed no significant trends. In contrast, in other regions (e.g., the coastal area of Paraíba State), the results were opposite when evaluating the SPI-48, which presented accentuated positive and negative Sen’s slopes based on SPI_gauge_ and SPI_TRMM_, respectively. Brasil Neto et al*.*^18^ found that the Pearson correlation coefficient between rain gauge-measured and TRMM-estimated rainfall data was negative in the coastal zones and positive in inland zones regarding the SPI-48 analysis, which supports the findings of the present study. The strong negative correlation between the datasets means that the trends move in opposite directions. Proximity to the coast, highest rainfall levels, the spatial distribution of the rain gauges, and the variability of the atmospheric mechanism, in the case of Mata Paraibana, were factors linked to the reduction of TRMM-estimated data accuracy^38,41^.

When performing an analysis of short-term droughts, the spatial variability of Sen’s slope over Paraíba State is highlighted. The results of SPI_gauge_ show that in the border region between Mata Paraibana and Agreste Paraibano, the trend of the time series is positive and significant, especially for the case of SPI-6. On the other hand, in the western portion of Agreste Paraibano and Borborema, the time series trend is negative. When analyzing the results of SPI_TRMM_, some inconsistencies can be highlighted. For SPI-1, most time series showed a trend with considerable significance, especially in Agreste Paraibano and Mata Paraibana. Furthermore, unlike the SPI_gauge_ results, the trends were predominantly negative in all mesoregions, highlighting the inconsistency between these results, mainly in regions close to the coast. It is worth noting that this inconsistency was as much to Sen’s slope as to Mann–Kendall’s statistical significance. However, Borborema showed negative trends using both datasets.

For medium-term droughts, Sen’s slopes are higher in the modulus than short-term droughts, and there are many series with a significant trend, regardless of the dataset. Based on SPI_gauge_, there is a considerable increase in the area with a significant trend (40% for SPI-9 and 50% for SPI-12) compared to those found for the SPI-1, which was 5%. Based on SPI_TRMM_, these percentage values were greater than 90% in SPI-9, and 100% when evaluating SPI-12, which reveals a high overestimation in the percentage of area with a significant trend. However, the spatial distribution of Sen’s slope is the same as that of short-term droughts, but these values are more accentuated. The results of SPI_gauge_ show that the border region between Agreste Paraibano and Borborema presents the greatest variation in trend slopes since the eastern portion has a positive trend, whereas the western portion presents a negative trend.

Although there is no significant change in the Sen’s slope in the Mata Paraibana, it is noteworthy that in the Sertão Paraibano, the trend slopes were more pronounced than the short-term droughts, and in most cases, the trends were negative. The results of SPI_TRMM_ show that the entire Paraíba State has negative trends, with emphasis on the Agreste Paraibano, Borborema and part of Mata Paraibana. For long-term droughts, the results are more extreme when dealing with the percentage of area with a significant trend and the Sen’s slopes. The SPI_gauge_ results show that the area with a negative trend at α ≤ 0.01 level was greater than 45%, which is higher than any other time scale. Furthermore, Sen’s slope values were higher than those of the other time scales, especially in the case of SPI-48; and the percentages of the area with the significant trend, obtained in this case, exceed three times the percentage in the case of short-term droughts.

The positive trends were concentrated in Mata Paraibana, in eastern Agreste Paraibano, and western Borborema. Negative values are in southwestern Sertão Paraibano, on the border between Agreste and Borborema and in Agreste’s central portion. It can be highlighted that the zone of greatest inconsistency between the results of SPI_gauge_ and SPI_TRMM_ occurred in the region close to the coast and that this is intensified when assessing the behavior of long-term droughts. Analyzing the results obtained by Brasil Neto et al.^18^, it is noted that this region has the lowest values of the correlation coefficient, indicating no satisfactory linear association between the SPI_gauge_ and SPI_TRMM_ time series.

From another point of view, Rao et al.^73^ evaluated the seasonal trends of rainfall over Brazil over 30 years and indicated that precipitation in the rainiest and driest periods over Paraíba State would increase, and events will tend to be wetter. Medeiros et al.^74^, in turn, evaluated the rainfall trends in the Sertão Paraibano (1912–2012) and concluded that rainfall has positive trends, especially when considering annual and semiannual time scales. Notoriously, these were not the results obtained in this study, and this may be related to the period of analysis used. It is important to point out that the data period influences the evaluation of trends. The data period used in the present research differs significantly from the periods used in those studies.

One of the most severe drought periods has recently occurred, which may have influenced the spatial distribution of Sen’s slopes. Marengo et al.^75^ pointed out that from the 21 most severe drought events that have occurred in NEB since 1900, six are included in the analyzed period of the present study, with particular emphasis on 1997–1998 and 2012–2015. This latest most recent event that reached the NEB caused approximately 1000 cities to declare a state of emergency and spurred social and economic conflicts. In this sense, one of the reasons that may have significantly influenced the present study's negative trends was this drought event during the recent years. In addition, as discussed by Páscoa et al.^76^ and Guo et al.^26^, the shorter the data period, the time series trend tends to be more significant, as is the case of the present study.

### Analysis of drought duration trends

**Figure 7:**
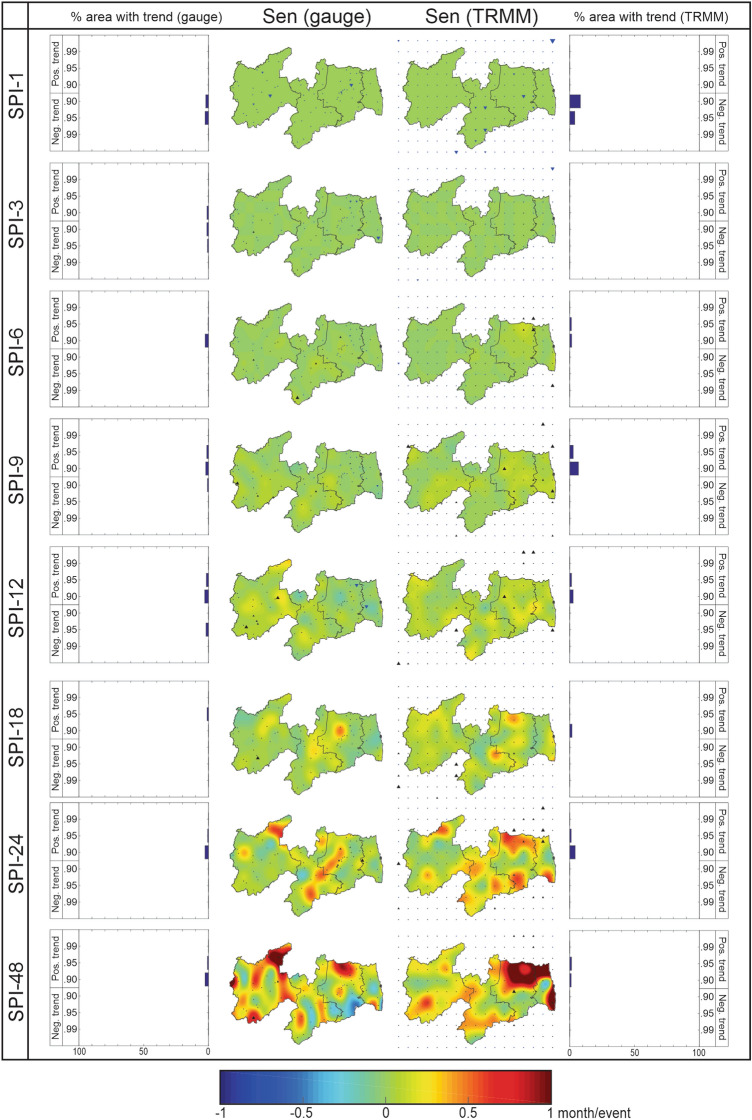
Percentage of the area with the significant trend and spatial distribution of Sen’s slope of the drought duration time series over Paraíba State (1998–2017).

Figure 7 shows the spatial distribution of the Sen’s slope of the drought duration time series and the percentage of area with a significant trend over Paraíba State at eight time scales. Regarding the pattern of the results considering the time scales, the most significant results of the Sen’s slopes were obtained for droughts at a large time scale. Regarding the percentage of area that tended to the most significant level of significance (α ≤ 0.01), the same pattern of results was not found for the drought behavior time series, as shown in Fig. 6. This result is strongly related to the amount of data in each time series submitted to trend analysis. As discussed by Santos et al.^59^, it is noted that a minimum amount of data in each time series is necessary for this to have relevant significance. For example, the minimum amount of data required for a time series to have significance at α = 0.010 is four, whereas, for significance to be α = 0.10, the time series must have at least seven data.

The amount of drought events decreases considerably when assessing long-term droughts, even though these events are more lasting and severe. For this reason, due to the reduction in the amount of data available for the duration time series, these time series may not have obtained a high significance level (e.g., α > 0.10), even though Sen’s slopes were relatively more accentuated. When comparing the results between the datasets, the results are even more similar to each other than the results of the behavior time series. However, it is noteworthy that, although it is not mandatory, the spatial distribution of Sen’s slope for the duration time series is also similar to the spatial pattern of Sen’s slope of the behavior time series.

It is worth arguing that it is not because the drought behavior time series has a positive trend, i.e., events tend to be wetter, that the duration time series will necessarily tend to decrease. Although the drought duration time series is derived from the behavior time series, these time series may show a negative trend, but the duration time series do not. When comparing the results of these figures, it is noted that there was an agreement between these time series in most cases. For SPI-48, for example, trends in drought behavior were negative, and trends in duration were positive in northeastern Agreste Paraibano, based on the rainfall data. Indeed, these results complement each other, as they indicate that events tend to be drier over time, and the duration of the drought events tends to be longer when these events occur.

When evaluating short-term droughts, it is noted that Sen’s slope values were not so significant and that only a few time series had a significant trend. These are mostly located in Agreste Paraibano and southwestern Sertão Paraibano in the case of SPI-1, in southern Mata Paraibana and northeastern Agreste Paraibano in the case of SPI-3, and southern Borborema in the case of SPI-6, based on SPI_gauge_ data. In addition, when evaluating SPI-6, the same pattern of Sen’s slopes was identified on the border between Agreste Paraibano and Borborema, and there are indications that drought events will be more lasting in this region. Based on SPI_TRMM_, Sen’s slopes were less accentuated for SPI-1 but more significant for SPI-6 and remarkably similar to SPI_gauge_ results. For statistical significance, there is an increase in the percentage of area with a significant trend compared to the results based on rain gauge-measured rainfall data, especially in southern Borborema for SPI-1, and in northeastern Agreste Paraibano for SPI-6.

According to Sen’s slope values and the low number of time series with significant trends, it can be said that for short-term droughts, the differences between the results obtained from SPI_gauge_ and SPI_TRMM_ are smaller when evaluating the drought duration time series than for the behavior time series. For medium-term droughts, the slopes are more pronounced, and the spatial variability is greater when compared to the results of short-term droughts. However, there is no change as relevant as the area with significant trends concerning short-term duration time series and the case of the behavior time series. For SPI_gauge_ results, there is a high percentage of significant trends when evaluating SPI-12 and a low percentage for SPI-9. Most of Mata Paraibana and Agreste Paraibano show negative trends, while Sertão Paraibano and Borborema have positive trends, which indicates that in these mesoregions, drought events tend to be more lasting.

The results of SPI_TRMM_ show positive trends throughout all mesoregions and indicate that the results found for medium-term droughts were similar to those for short-term droughts. There was an inconsistency between the two datasets in the Mata Paraibana mesoregion and on the border between Agreste Paraibano and Mata Paraibana. However, these results were less discrepant than those obtained in the analysis of the behavior time series. As for the duration trends of drought events, it is noted that the results obtained in this research corroborate with those obtained by Awange et al.^21^, who also showed that drought events tend to be more lasting over Paraíba State. However, in the north of the NEB, the trend is that drought events will become less lasting.

Finally, for long-term droughts, the highest and lowest Sen’s slope values are found among the time scales, although the area with a significant trend is reduced compared to the results obtained in Fig. 6. The results found for SPI_gauge_ indicate that Agreste Paraibano and Borborema have positive trends when evaluating the SPI-18 and SPI-24. For the SPI-48, in turn, these slopes tend to be even more pronounced, especially in northeastern Agreste Paraibano and in northern Sertão Paraibano. In these regions, the slope values indicate an increase of 1 month per drought event; i.e., future drought events tend to last for a month. In the southeastern Agreste Paraibano and on the coast of Paraíba State, on the other hand, Sen’s slopes are negative and accentuated, indicating that drought events will last less.

In comparison with the SPI_TRMM_ results, it is noted that there is an agreement between the results, especially in the case of SPI-18. For SPI-24, the trend slopes in northeastern and southeastern Agreste Paraibano and the central region of Borborema were overestimated. For the SPI-48, the trend values were more pronounced in northern Agreste Paraibano and the southeastern coast of Paraíba State. On the other hand, it is interesting to note that in the central portion of Mata Paraibana, there was coherence on the part of the satellite-estimated rainfall data when detecting negative trends in the duration of drought events, corroborating with the results found based on SPI_gauge_ data. However, in northern Mata Paraibana, in southeastern Agreste Paraibano, and in large part of Borborema, TRMM-estimated rainfall data did not precisely identify the trend patterns of the drought duration time series.

### Analysis of drought severity trends

**Figure 8:**
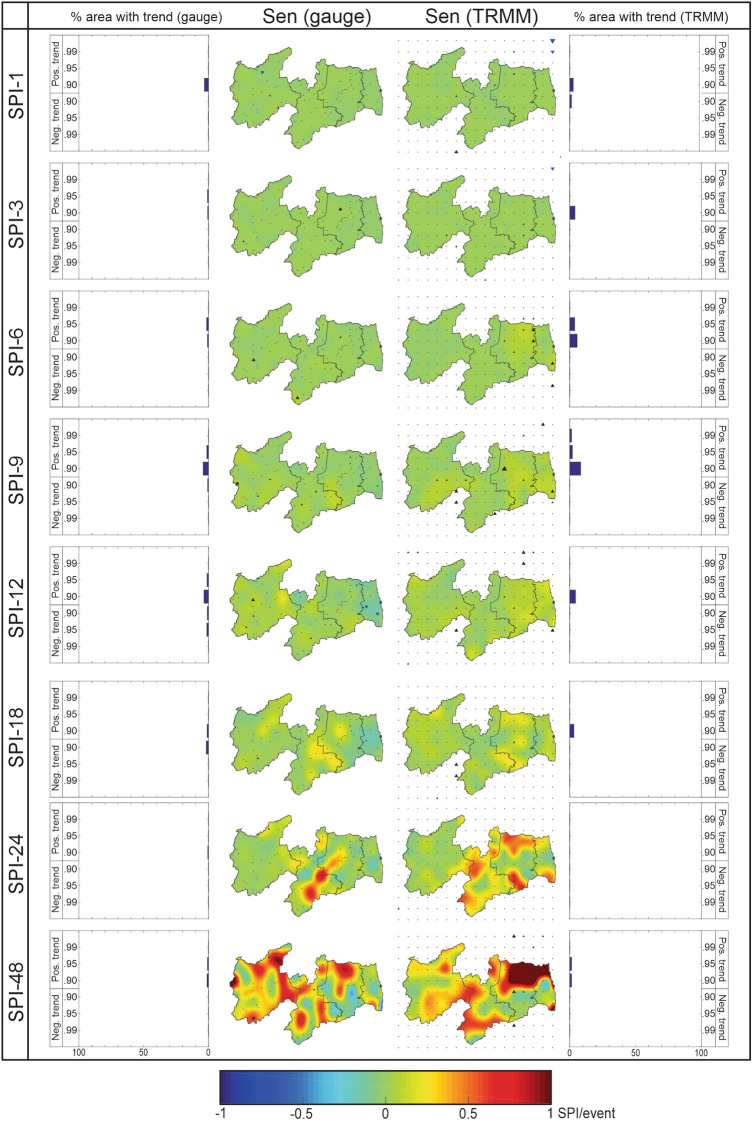
Percentage of the area with the significant trend and spatial distribution of the Sen’s slope of the drought severity time series over Paraíba State (1998–2017).

Similar to the results of Figs. 6 and 7, Fig. 8 shows the spatial distribution of the Sen’s slope of the drought severity time series and the percentage of area with a significant trend over Paraíba State at multiple time scales. In general, there is considerable similarity between the results of the time series of drought duration and severity, in terms of the levels of significance, in the Sen’s slopes and the SPI_gauge_ and SPI_TRMM_ results. In the case of short-term droughts, the slopes are less pronounced, which was also observed in the analyses of drought duration and behavior time series. In addition, it is interesting to note that in some cases, such as southern Borborema and northeastern Agreste Paraibano, in the case of the SPI-3 and SPI-6, the trend is significantly positive both when assessing severity and duration.

This information is relevant because it provides evidence of the trend in the intensity of the drought events. In general, if the slopes of the drought duration and severity time series are the same, this probably implies that the intensity of drought events tends to remain constant over time. On the other hand, if the duration time series has a negative trend, i.e., the events tend to be less lasting, and the severity time series has a positive trend, i.e., the events tend to be more severe, it is possible to state that the intensity of the drought events will increase. The result highlights the importance of conducting a joint assessment of the trend analysis of these three types of time series, as one can understand the pattern of drought trends not only in terms of behavior (Fig. 6) but also in terms of duration (Fig. 7), severity (Fig. 8), and indirectly as in terms of intensity.

When using SPI_TRMM_, the percentage of area with a significant trend is higher than that obtained for SPI_gauge_ results. For SPI-6, for example, the percentage using SPI_TRMM_ is four times higher than the values using SPI_gauge_, which were concentrated in Agreste Paraibano and Mata Paraibana. The trends were predominantly positive when considering the two datasets. It can be said that the disagreements between the SPI_gauge_ and SPI_TRMM_ results were smaller compared to those obtained in the drought behavior time series. In the case of medium-term droughts, the slope values increased in modulus, and an area started to show a trend with relevant significance, as is the case of the drought duration time series. Based on SPI_gauge_ data, the trends were positive in southern Borborema and in Sertão Paraibano, revealing an increase in the severity of the drought events.

On the other hand, close to the coast, the slope was negative mainly for SPI-12, which reveals that drought events will be less severe. Based on the SPI_TRMM_ results, there was an increase in the percentage of area with a significant trend concerning the results obtained for short-term droughts. Except for isolated areas in Sertão Paraibano and Borborema, most grids showed a positive trend, as found in the case of drought duration time series. Once again, Agreste Paraibano and Mata Paraibana were the mesoregions where the severity time series presented the most accentuated positive slopes. This situation exposes the discrepancy between the results obtained based on rain gauge-measured rainfall data. In addition, in comparison with the results of the duration time series, it is noted that the severity time series showed less expressive slopes when evaluating the SPI-12. This indicates that, although the duration and severity time series have positive trends, the first one had more accentuated slopes than those of severity.

As the duration tends to increase at a rate greater than severity, it is estimated that drought events will be less intense, although they tend to be more lasting and severe. Finally, for the analysis of long-term drought events, the most pronounced Sen’s slopes among the analyzed scales were observed, a result that is similar to that found in Figs. 6 and 7, and to the studies developed in Asia^20^ and Africa^22^. It is worth noting that the results of trends in duration and severity are similar to each other, but some caveats will be discussed. For SPI-18, there is a drop in the magnitude of the slope compared to the duration time series results, and the same occurs in northern and western Sertão Paraibano in the case of SPI-24, and in eastern Mata Paraibana in the case of SPI-48.

In all situations, as well as for SPI-12, it is estimated that drought events will tend to be less intense, as the slope of duration time series tends to be higher than the slope of severity time series. On the other hand, in northeastern and southeastern Agreste Paraibano, the situation is reversed; e.g., the severity time series present steeper slopes than the duration time series, in the case of SPI-48. The results of SPI_gauge_ show that the severity of drought events in Mata Paraibana and Agreste Paraibano tends to fall, but in Sertão Paraibano and Borborema, the trends are positive. When evaluating the SPI_TRMM_ results, almost the entire Paraíba State starts to show positive trends. The highest values are found in northern and northeastern Agreste Paraibano and Mata Paraibana and the drought duration time series.

In these regions, the slope values exceed the rate of 1 unit of SPI per drought event. In other words, this implies that based on the drought events that happened over the 20 years evaluated, the next events tend to have severity values plus one unit, which reveals a considerable increase compared to the results of the short-term droughts. For the precision of TRMM-estimated data in capturing Sen’s slopes and the percentage of area with a significant trend, the similarity with the results obtained in the analysis of trends in drought duration is highlighted.

### Trend analysis per mesoregion

**Figure 9:**
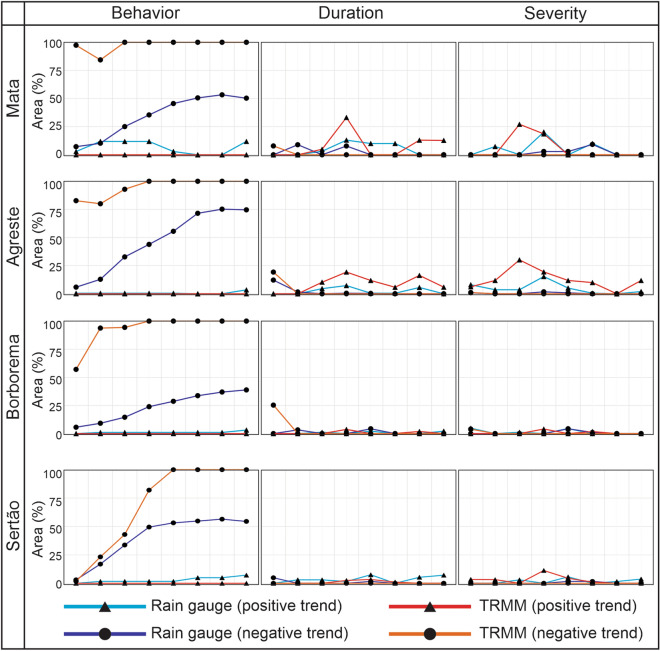
Analysis of the percentage of area with a significant trend based on the time series of drought behavior, duration and severity for the mesoregions of Paraíba State (1998–2017).

Finally, to carry out an analysis for each mesoregion, Fig. 9 shows the percentage of area with a significant trend (α ≤ 0.10) for the drought behavior, duration and severity time series for the mesoregions of Paraíba State. The results indicate that the percentage of area with a significant trend that had the greatest variability was obtained when evaluating the drought behavior time series. In contrast, in the case of the drought duration and severity time series, this variation was not accentuated. It was also observed that the temporal scale considerably influenced the variation of the percentage of area with a significant trend, such that the values were significant when evaluating long-term droughts, regardless of the mesoregion or dataset.

Mata Paraibana has a substantial inconsistency between the SPI_gauge_ and SPI_TRMM_ results, mainly when assessing the drought behavior time series. Although both datasets have identified that Mata Paraibana has a predominantly negative trend, the percentages were notoriously overestimated by SPI_TRMM_. However, this difference was smaller when dealing with long-term droughts (i.e., SPI-18, SPI-24 and SPI-48). For duration and severity time series, the SPI_TRMM_ overestimation was related to the percentage of area with positive trends, but the discrepancies were smaller than the results obtained for the behavior time series. The highest percentage values for duration and severity were obtained when evaluating SPI-9 and SPI-12, showing consistency between SPI_gauge_ and SPI_TRMM_ results.

In Agreste Paraibano, one can note a more evident similarity between the SPI_gauge_ and SPI_TRMM_ results when compared to those of Mata Paraibana. Although there is an overestimation in the percentage of area with a negative trend on the part of the satellite-estimated rainfall data, it is noteworthy that this fact occurred to a lesser extent than the pattern of Mata Paraibana. In general, when analyzing the drought behavior time series, the results were less accurate for short- and medium-term droughts than for long-term droughts, whose percentage of area with a negative trend was greater than 75% for both datasets. For the drought duration and severity time series, the area percentage with a significant trend is less than 25%. It reaches the maximum values in the case of SPI-9 for the duration time series and SPI-6 for the severity time series.

In the Borborema mesoregion, it is noted that although the results obtained between the two datasets were remarkably similar in the case of the duration and severity time series, in the case of the behavior time series, there is a notable inconsistency between the percentage values obtained from the rain gauge-measured and TRMM-estimated rainfall data. The satellite-estimated data identified that the pattern of trends was predominantly negative, but the area's overestimation was greater than 50% in most cases. In Sertão, there are similarities between the results of the two datasets, which occurred when assessing the drought behavior, duration and severity. The percentage of area with a significant trend obtained from the two datasets was very similar when evaluating the short- and medium-term drought behavior time series. For the long-term time series, there was an overestimation of the TRMM-estimated data.

## Conclusions

From this study, the behavior and trends of multiple-scale droughts over Paraíba State using rain gauge-measured and satellite-estimated rainfall data were evaluated, and the performance of TRMM-estimated rainfall data to capture rainfall and drought patterns was very satisfactory. The satellite-estimated rainfall data accurately identified one of the events categorized as one of the most severe droughts in recent times. It was possible to observe that when evaluating long-term droughts, the percentage of dry events decreased in frequency but increased in severity.

For the trend analysis, it was noted that the larger the time scale, the more significant were Sen’s slopes and the significance of the behavior time series. In the case of the duration and severity of droughts, although it is clear that the events tended to be more lasting and severe with the increase in the time scale, the significance of these time series was not sensitive to the variation of the time scale as well as in the case of the behavior time series. Regarding the pattern found among the mesoregions, the events tended to be drier, longer-lasting, and more severe in most of the state. The greatest inconsistencies between the results obtained based on rain gauge-measured and TRMM-estimated rainfall data are concentrated in the area closest to the coast of Paraíba State.

The results obtained using the TRMM-estimated rainfall data identified trends in Paraíba State. This was more precisely when assessing the duration and severity of drought events. Finally, it is concluded that the TRMM-estimated rainfall data are a valuable source of data to identify the drought patterns and trends in large part of Paraíba State at multiple time scales and, therefore, the results obtained may contribute to the drought monitoring in similar regions worldwide. Additionally, we recommend that more studies involving other satellite products and drought indices should be done over Paraíba State and other similar regions to improve the preparedness for future drought hazards using remote sensing data.
